# Associations between work ability and work participation after rehabilitation: a longitudinal multicentre cohort study

**DOI:** 10.1016/j.ero.2026.03.011

**Published:** 2026-04-17

**Authors:** Mari Nilsen Skinnes, Till Uhlig, Thomas Johansen, Idun Eid, Hanne Ludt Fossmo, Andreas Habberstad, Katerina Kandlova, Ingvild Kjeken, Tarja Rajalahti Kvalheim, Peter Solvoll Lyby, Ross Wilkie, Nina Østerås, Rikke Helene Moe

**Affiliations:** 1 Center for Treatment of Rheumatic and Musculoskeletal Diseases (REMEDY), Diakonhjemmet Hospital, Oslo, Norway; 2 Institute of Health and Society, Faculty of Medicine, University of Oslo, Oslo, Norway; 3 Norwegian National Advisory Unit on Occupational Rehabilitation, Rauland, Norway; 4 Viker Helse, Kapteinveien, Hønefoss, Norway; 5 Vikersund Rehabilitation Centre, Vikersund, Norway; 6 The Norwegian Federation of Organisations of Disabled People, Oslo, Norway; 7 Red Cross Haugland Rehabilitation Centre, Flekke, Norway; 8 CatoSenteret Rehabilitation Centre, Son, Norway; 9 Keele University, Keele, Newcastle, UK

## Abstract

**Objectives:**

To examine whether work ability at 3 and 12 months after rehabilitation admission was associated with work participation, operationalised as health-related benefit use in the year following rehabilitation.

**Methods:**

This prospective longitudinal, multicentre cohort included 2710 Norwegian working-age adults undergoing rehabilitation. The majority had rheumatic and musculoskeletal diseases (42%). Work ability was self-reported using the Work Ability Score at 3 and 12 months after admission. The number of days on health-related benefit sick leave, work assessment allowance, and disability benefits in the year before rehabilitation (year −1); the rehabilitation year (year 0); and the following year (year 1) were extracted from national registries. Associations between work ability and benefit use were examined in linear regression models adjusted for baseline work ability, health and sociodemographic factors, and prior health-related benefit use.

**Results:**

Higher work ability at 3 and 12 months was associated with fewer days on work assessment allowance in year 1, with −9.5 (95% CI: −11.9, −7.2) and −10.4 days (95% CI: −12.2, −8.7), respectively. Similarly, higher work ability was also associated with fewer days on disability benefits, with −5.0 days (95% CI: −6.7, −3.3) and −6.3 days (95% CI: −7.9, −4.5), respectively. Higher work ability at 3 months, but not at 12 months, was associated with 1.5 additional sick leave days (95% CI: 0.8, 2.3) in year 1.

**Conclusions:**

Postrehabilitation work ability was associated with subsequent work participation, reflected in lower use of health-related benefits. Assessing work ability after rehabilitation may indicate future work participation.

**WHAT IS ALREADY KNOWN ON THIS TOPIC**

 

• Rheumatic and musculoskeletal diseases are a major cause of sick leave and work disability
• Rehabilitation aims to promote functioning, coping, and participation, including participation in work. However, its effects on work participation have mainly been studied in small, selected populations, with limited evidence in larger, heterogeneous rehabilitation populations
• Work ability is associated with future sick leave and disability, but how it relates to the use of health-related benefits after rehabilitation in heterogeneous populations remains unclear

**WHAT THIS STUDY ADDS**

 

• Higher work ability after rehabilitation was associated with improved work participation, demonstrated by lower use of work assessment allowance and disability benefits in the following year
• Assessment of work ability can identify people at risk of future labour market exclusion in heterogeneous rehabilitation populations

**HOW THIS STUDY MIGHT AFFECT RESEARCH, PRACTICE OR POLICY**

 

• Routine assessment of work ability can help guide rehabilitation interventions to improve long-term work participation
• Work ability assessment can identify patients likely to rely on health-related benefits, informing workplace, health strategies, and policy planning

Alt-text: Unlabelled box dummy alt text

## INTRODUCTION

Noncommunicable diseases are a leading cause of disability and reduced work participation [1]. Rheumatic and musculoskeletal diseases (RMDs) account for a large proportion of long-term sick leave and work disability, and their prevalence is expected to rise in the coming decades, partly due to an ageing population [[2], [3], [4]]. People with chronic conditions, including RMDs, have lower levels of work participation than the general population, with nearly 40% of working-age adults with disabilities in the European Union (EU) remaining outside the labour market [3,5,6]. Staying in, or returning to, work can provide financial security, social connection, a sense of purpose, societal contribution, and health benefits [[7], [8], [9], [10]].

Rehabilitation programmes aim to help people maintain or regain health and work ability [9,11]. Work participation is a key objective in the Norwegian definition of rehabilitation, which defines it as an individualised, goal-oriented process that seeks to promote functioning, coping, and participation, including participation in education, work, and social life [12]. Such programmes typically address physical, psychological, and occupational barriers, and may include (psycho)education, cognitive-behavioural therapy, psychosocial support, physical exercise, and group and vocational counselling, with generally small but positive effects on work participation [9,13].

Work ability reflects the balance between job demands and personal resources, and it changes over the course of working life [14]. By assessing work ability in a rehabilitation setting, health professionals may identify people’s needs, guide tailored rehabilitation programmes, and evaluate outcomes [15]. The Work Ability Score (WAS) measures work ability by asking people to compare their current work ability with their lifetime best.

Previous research has shown that the WAS predict both self-reported and registry-based sick leave and disability pension in various working populations [[16], [17], [18], [19]]. However, the association between the WAS after rehabilitation and work participation has not been explored in a rehabilitation population with differing diagnoses and health-related benefit types.

The objective of this study was to examine whether work ability at 3 and 12 months after rehabilitation admission was associated with work participation, operationalised as health-related benefit use in the year following rehabilitation.

## METHODS

### Study design, participants, and interventions

This study is part of *RehabNytte*, a large, prospective, longitudinal, multicentre rehabilitation cohort with a 12-month follow-up. Seventeen rehabilitation institutions across Norway participated in data collection. Data were gathered at admission, discharge, and at 3-, 6-, and 12-month postadmission [20]. Additionally, registry data from the Norwegian Labour and Welfare Administration (Nav) on health-related benefit use were extracted for the year before rehabilitation (year −1), the rehabilitation year (year 0), and the year after rehabilitation (year 1). Participants were recruited at the participating institutions by local project coordinators from January 2019 to March 2020, and the last follow-up data collection was completed in June 2021.

All patients referred to one of the participating rehabilitation institutions were eligible. Referrals were made by general practitioners or medical specialists and coded according to ICD-10. Referral to rehabilitation was based on functional problems or risk of reduced functioning, whereas the referral diagnosis reflects the primary medical condition underlying these problems.

Inclusion criteria were admission to a participating rehabilitation institution, age 18 years or older, adequate Norwegian language skills, and internet access. Patients were excluded if they had a history of head injury with impaired cognitive functioning or other conditions preventing informed consent.

Only participants of working age (≤65 years), who were not retired and consented to data collection on work and benefit status, were included in the current analyses. No à *priori* power calculations were performed due to the large sample size.

### Rehabilitation intervention

Rehabilitation programmes were tailored to each patient by multidisciplinary teams and varied in structure (inpatient, outpatient, and mixed settings due to geographical constraints), goals, content and duration (ranging from 1 week to 6 months), dependent on individual needs [21] (Supplementary materials, Textbox: Brief description of the rehabilitation programmes). All 17 institutions provided multidisciplinary rehabilitation, and 9 additionally offered occupational rehabilitation.

### Sickness insurance system

Norway’s national insurance system provides income replacement (benefits) to cover lost earnings during illness, and all residents are covered by this public scheme.

#### Sick leave (sickness benefits)

Employees who are fully or partially absent from work due to temporary illness or injury, as reported by authorised health professionals, are entitled to sick leave. The degree of sick leave, graded from 20% to 100%, is usually determined during a consultation with the patient’s general practitioner. Employees receive 100% wage compensation for up to 52 weeks, with the first 16 days paid by the employer and the remainder by Nav. After 52 weeks, people may transition to work assessment allowance (WAA).

#### WAA

People with at least 50% reduced work ability, albeit with possible return to work determined by Nav, are eligible for WAA. Recipients must participate in medical follow-up and work-related activities. WAA can be combined with part-time work, but working more than 60% may disqualify recipients. It is available for up to 3 years, with possible extensions. WAA is taxable and count towards pension allowances.

#### Disability benefits

People with permanent work incapacity of at least 50% are eligible for disability benefits. Such benefits can be combined with part-time work; if earnings exceed 80% of previous income, benefits are withheld. Disability benefits are granted until retirement age, are taxable, and counts towards pension allowances.

Both WAA and disability benefits typically correspond to up to 66% of previous income [22]. Because these benefits compensate for full-time or part-time work, people may receive more than 1 type of benefit concurrently: eg, receiving 50% disability benefits while being sick-listed for the remaining work capacity.

### Ethical considerations and patient engagement

The regional ethical committee determined that formal approval was not required for the RehabNytte study, given its primary focus on evaluating rehabilitation service delivery (2018/1645/REK Sør-Øst A). The study was approved by the data protection officer at Diakonhjemmet Hospital (DS-00040) on October 17, 2018, and registered at ClinicalTrails.gov (NCT03764982). All participants received oral and written information about the study and provided written informed consent in accordance with the Declaration of Helsinki [23].

Two experienced patient research partners were actively involved throughout the study as coresearchers, and contributed to study design, survey materials, interpretation of results, manuscript preparation, and dissemination of results. Their involvement strengthened the relevance, clarity, and accessibility of the study [24].

### Primary outcomes

The study included 4 primary outcomes, all reflecting work participation. These were the total number of days on sick leave, WAA, and disability benefits, calculated per person, during the year following rehabilitation (year 1), as well as a combined measure of total benefit days. Data were extracted from Nav’s registry, and all time periods were related to the initiation of rehabilitation given by the admission date. To account for employment fractions, such as combining work with part-time sick leave, graded WAA, or partial disability while working, the benefit days were converted, reflecting *compensated full workdays*.

### Sociodemographic and health-related factors

Work ability was self-reported at admission to rehabilitation (baseline), and again 3 and 12 months later (year 0) using the WAS, a single-item measure ranging from 0 (completely unable to work) to 10 (work ability at its best) [25,26]. This item is derived from the Work Ability Index and has not been separately validated in Norwegian but in Swedish among working-age patients with chronic pain [27]. Given the closely related languages and cultural similarity between Sweden and Norway, this validation is considered relevant in the Norwegian context. The WAS correlates strongly with the full index and has demonstrated good measurement properties and prognostic accuracy [[27], [28], [29], [30]].

Sociodemographic variables were self-reported and included age, gender, and marital status. The term gender, rather than ‘sex’, was used as it encompasses the social and cultural aspects of how people perceive themselves [31]. The geographical region was determined by the location of the rehabilitation institution within 1 of Norway’s 4 main health regions (South-East, West, Mid, and North). Regions ‘Mid’ and ‘North’ were combined in the analysis due to fewer inhabitants and participating rehabilitation institutions in the Northern region. The education level variable was dichotomised into higher (>12 years of education) vs lower level (≤12 years). Occupational background was grouped into 2 categories: manual occupations (including unskilled, apprentice, craftsman, and subordinate roles) and nonmanual occupations (including professional, academic, and leadership positions). The type of rehabilitation programme was categorised as multidisciplinary or occupational rehabilitation (Supplementary materials: Textbox).

Health-related factors at baseline included the primary referral diagnosis, defined as the main complaint for which rehabilitation was prescribed, assigned by the referring physician and categorised into 3 groups: RMDs, cancer or other conditions to ensure sufficient numbers within each category to allow meaningful statistical analyses. The ‘other’ category comprised less frequent diagnoses such as neurological, cardiological, and pulmonary conditions, as well as lifestyle-related disorders such as diabetes and obesity. Self-reported health status included comorbidities from a list of 19 diseases. The number of diseases was then summarised and subgrouped (none, 1-2, or ≥3). Self-reported body weight and height at baseline were used to calculate the body mass index (kg/m^2^). Other self-reported health variables included smoking status (never, sporadic, daily), widespread pain (yes/no), and pain intensity (Numeric rating scale]: 0-10). Anxiety and depression were assessed using a single item from the EuroQoL 5D, originally a 5-level ordinal scale, which was dichotomised into ‘No or Slight’ and ‘Moderate to Extreme’ [32]. A dichotomous variable indicated whether data collection on an individual level occurred during the COVID-19 pandemic, with dates after March 15, 2020 marking the pandemic period. The dichotomous coding (yes/no) was chosen to capture the major societal and service-related disruptions caused by the pandemic, while keeping the analysis clear and interpretable.

### Statistical analyses

Descriptive statistics of demographic and baseline variables are presented as proportions, means and SD, or medians and range, as appropriate. The development of benefits over the 3-year period was visualised using line plots and bar charts.

To visualise the association between the WAS at 3 and 12 months after rehabilitation admission (year 0) and total days on benefits in the year following rehabilitation (year 1), we created combined scatter and cumulative probability plots. Each person’s total benefit days were plotted against their cumulative frequency, alongside their WAS on a secondary y-axis, illustrating the relationship between benefit use and work ability.

To examine associations between work ability 3 and 12 months after rehabilitation admission (year 0) and health-related benefit use in the year following rehabilitation (year 1), linear regression models were fitted for each of the 4 primary outcomes (sick leave, WAA, disability benefits, and total benefit days). WAS at 3 and 12 months was analysed in separate models adjusted for the baseline WAS. Model assumptions (normality and homoscedasticity of residuals) were checked, and minor deviations were observed. Given the large sample size (N = 2710), the method was considered robust to these deviations [33].

The following analyses proceeded in 3 steps. First, bivariate linear regression analyses were used to examine crude associations between independent variables of interest and each primary outcome.

Second, variables with *P* < .20 in the bivariate analyses were entered separately into models adjusted for key covariates. These key covariates included age, gender, diagnosis group, subgrouped number of comorbidities, and geographic region, as well as days on sick leave, WAA, and disability benefits in the year prior to rehabilitation (year −1). The key covariates were selected à *priori* based on their clinical relevance as stated in the literature [34]. Using a higher threshold in bivariate screening is in line with recommendations to avoid excluding potentially relevant factors [35]. Finally, all independent variables with *P* < .20 and key covariates were simultaneously entered into multivariable models. Then, a backward stepwise regression approach was used, in which independent variables were removed stepwise from the multivariable models if *P* > .05, to obtain parsimonious final models. Key covariates were retained in all models regardless of statistical significance. Results were reported as beta coefficients with 95% CI. For consistency, only independent variables that were statistically significant in both the bivariate and final multivariate models are presented Table 1.

**Table 1:** Baseline characteristics

Characteristic	All (N = 2710)
Age, y, mean (SD)	47.9 (10.3)
Gender, female n (%)	1958 (72.3)
Diagnosis, n (%)	
Cancer	584 (21.6)
Rheumatic and musculoskeletal diseases	1130 (41.9)
Other	986 (36.5)
Cardiovascular	53 (2.0)
Lifestyle/obesity	289 (10.7)
Neurological	344 (12.8)
Sensory impairments	109 (4.0)
Mental	63 (2.3)
Other	128 (4.7)
Comorbidities, n (%)	
None	408 (15.1)
1-2	1451 (53.5)
3 or more	851 (31.4)
BMI, kg/m^2^, median (25 and 75 percentile)	28.4 (24.6, 33.8))
Smoking, n (%)	
Never	993 (42.7)
Sporadic	1103 (47.4)
Daily	231 (9.9)
Married/cohabiting, n (%)	1381 (59.2)
Geographic region, n (%)	
West	393 (14.5)
South-East	2047 (75.5)
North and Mid-Norway	270 (10.0)
High education level (>12 y), n (%)	1068 (45.9)
Work Ability Score (0-10), mean (SD)	3.2 (2.9)
Current or last main work, n (%)	
Manual occupations	1399 (60.9)
Nonmanual occupations	900 (39.2)
Widespread pain, yes, n (%)	1411 (74.1)
Pain intensity (0-10), mean (SD)	4.7 (2.4)
Anxiety/depression (single item from EQ-5D-5L)	
No or slight	1653 (72.0)
Moderate to extreme	643 (28.0)
Days with benefits in the year before rehabilitation	
Sick leave (mean, SD)	46.1 (63.0)
Work assessment allowance (mean, SD)	58.2 (96.0)
Disability benefits (mean, SD)	46.8 (92.8)
Occupational rehabilitation, n (%)	575 (21.2)

BMI, body mass index; EQ-5D-5L, EuroQoL 5D.

Analyses were conducted using available case data, with no imputation for missing values. All analyses were performed using StataCorp LLC, USA (STATA) statistical software, version 18 [36], with a significance level set at *P* < .05.

## RESULTS

Of 3731 eligible participants, 2710 met the inclusion criteria and were included. The mean age was 48 years, and 72% were female (Table 2). The primary diagnoses were: RMDs (42%), cancer (22%), and other conditions (36%). Most participants (85%) had 1 or more comorbidities. The mean (SD) baseline pain intensity was 4.7 (2.4), with 70% reporting pain in multiple locations. The mean WASs at baseline, 3, and 12 months were 3.2, 4.2, and 4.6, respectively. In the year prior to rehabilitation (year −1), participants had on average 46 days on sick leave, 58 days on WAA, and 46 days on disability benefits.

**Table 2:** Associations between total benefit days in the year after rehabilitation (year 1) and work ability at 3 months after rehabilitation admission (year 0)

Variable	Bivariate model β, (95% CI)	*P* value	Multivariate model^a^ β, (95% CI) *R*^2^ = .59	*P* value
Work Ability Score 3 mo	-23.4 (-24.5, -22.2)	<.001	-12.6 (-14.3, -10.9)	<.001
Work Ability Score baseline	-21.0 (-22.2, -19.7)	<.001	-3.1 (-4.9, -1.3)	.001
Smoking				
Never	Ref.		-	
Sometimes	15.3 (6.1, 24.6)	.001	-	
Daily	32.5 (17.0, 47.9)	<.001	-	
BMI	-1.6 (-2.3, -1.0)	<.001	-	
Education				
≤12 y	Ref.		Ref.	
≥13 y	-35.8 (-44.5, -27.1)	<.001	-19.2 (-26.0, -12.3)	<.001
Current or last main work			-	
Manual occupations	Ref.		-	
Nonmanual occupations	-33.1 (-42.1, -24.1)	<.001	-	
Widespread pain				
No	Ref.			
Yes	42.8 (32.1, 53.5)	<.001	-	
Pain intensity	10.4 (7.7, 13.1)	<.001	-	
Anxiety/depression				
No or slight	Ref.			
Moderate to extreme	32.3 (22.5, 42.1)	<.001	-	
Occupational rehabilitation				
No	Ref.			
Yes	-6.4 (-16.3, 3.6)	.2	-	
Sick leave in the year before rehabilitation	-0.2 (-0.3, -0.1)	<.001	0.3 (0.2, 0.3)	<.001
Work assessment allowance in the year before rehabilitation	0.4 (0.4, 0.4)	<.001	0.4 (0.3, 0.4)	<.001
Disability benefits in the year before rehabilitation	0.5 (0.4, 0.5)	<.001	0.5 (0.4, 0.5)	<.001

BMI, body mass index; Ref., reference category.

a Multiple linear regression with backward selection, adjusted for age, gender, geographic region, comorbidities, and diagnoses.

**Figure 1:**
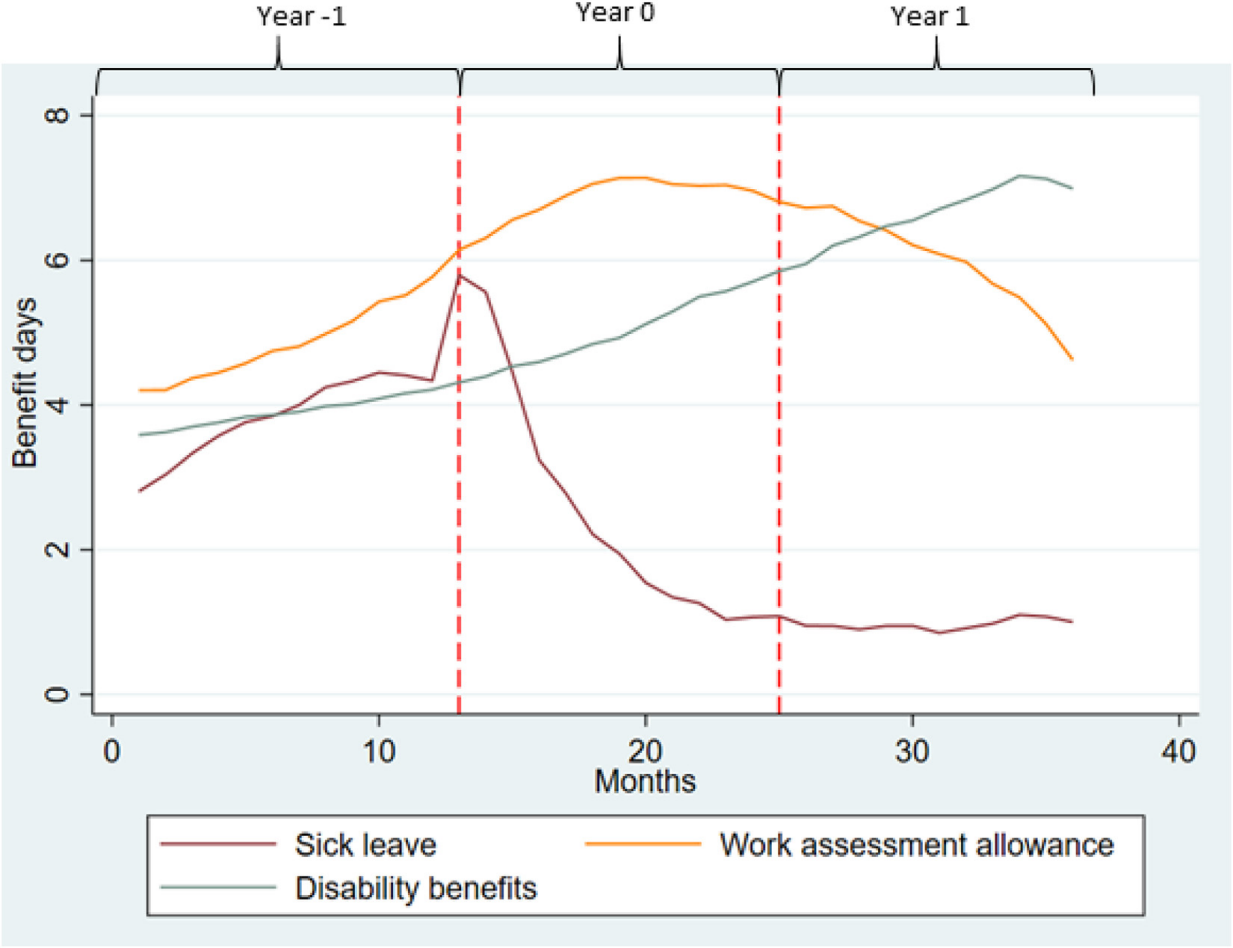
Mean days on benefits per month during the 3-year period. The 2 red dashed lines indicate the rehabilitation admission, and the following 12 months (year 0), respectively.

During the 3-year period, sick leave rose at the time of rehabilitation admission, then declined in the rehabilitation year (year 0) and remained low in the following year (year 1) (Fig 1). WAA increased in the year before rehabilitation (year −1), reached a maximum during year 0, and declined again in year 1. Disability benefits increased gradually throughout the 3-year period. Line plots of days on benefits based on the type of rehabilitation are shown in Supplementary Figures S1 and S2.

**Figure 2:**
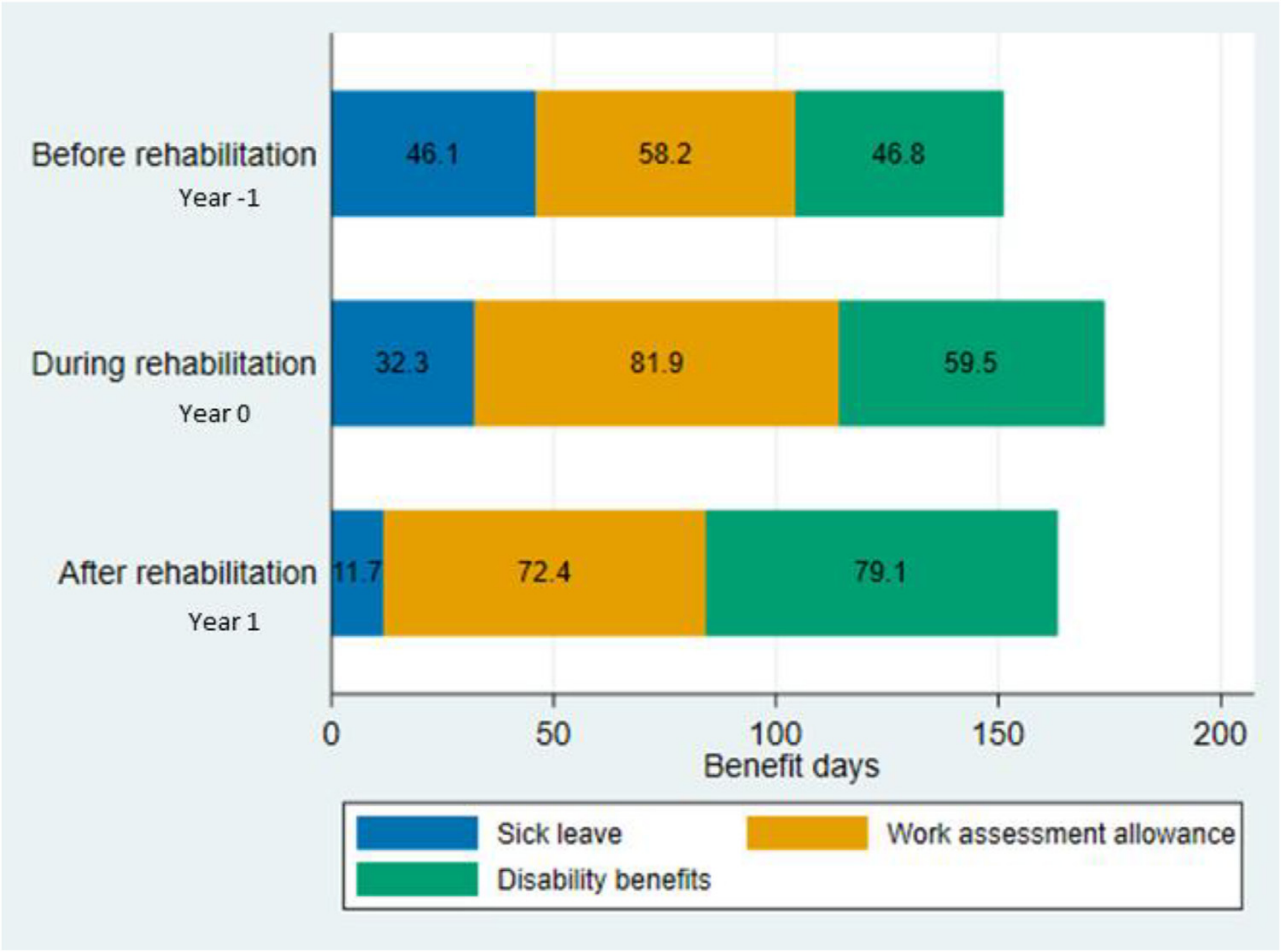
Mean days on sick leave, work assessment allowance, and disability benefits over the 3-year period.

Figure 2 shows the mean number of days on the 3 types of benefits over the 3-year period.

**Figure 3:**
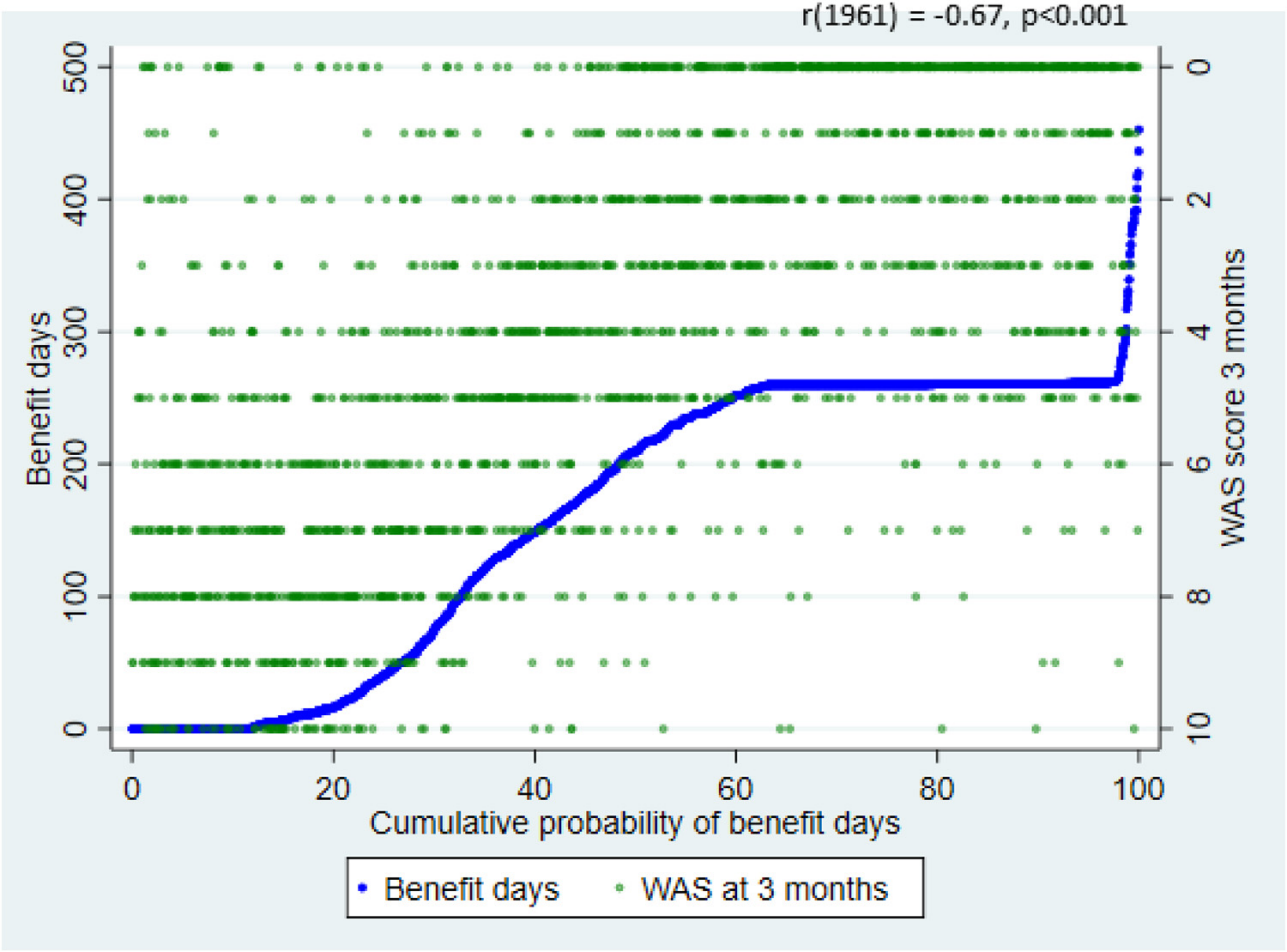
Relationship between Work Ability Score (WAS) at 3 months after rehabilitation admission (year 0) and number of benefit days in the year after rehabilitation (year 1) in a cumulative probability plot. Each dot represents 1 patient.

We plotted the relationship between work ability at 3 and at 12 months after rehabilitation admission (year 0) against benefit days in the year following rehabilitation (year 1) using a combined scatter and cumulative probability plot (Fig 3). The blue dots represent the number of benefit days, with more days among participants reporting lower work ability. The bottom right corner illustrates that few participants with high work ability were among those with many benefit days, whereas the top left corner shows that few participants with low work ability had few days on benefits (Fig 3 and Supplementary Fig S3). Sixty per cent of participants had fewer than 250 days on benefits, and the median was 200 days.

Work ability at 3 months after admission to rehabilitation was significantly associated with benefit use in the year after rehabilitation (year 1) (Table 3). A 1-point increase in work ability was associated with 12.6 fewer days on benefits (95% CI: −14.3, −10.9). Higher education was associated with 19.2 fewer days on benefits (95% CI: −26.0, −12.3) (Table 3).

**Table 3:** Associations between sick leave, work assessment allowance, and disability benefits in the year following rehabilitation (year 1) and work ability at 3 months after rehabilitation admission (year 0)

	Sick leave model				Work assessment allowance model				Disability benefits model			
Variable	Bivariate model β (95% CI)	*P* value	Multivariate model^a^ β (95% CI) *R*^2^ = .14	*P* value	Univariate model Β (95% CI)	*P* value	Multivariate model^a^ β (95% CI) *R*^2^ = .33	*P* value	Univariate model Β (95% CI)	*P* value	Multivariate model^a^ β (95% CI) *R*^2^ = .66	*P* value
Work Ability Score 3 mo (NRS 0-10)	2.6 (2.2, 3.0)	<.001	1.5 (0.8, 2.3)	<.001	-10.0 (-11.4, -8.7)	<.001	-9.5 (-11.9, -7.2)	<.001	-15.9 (-17.3, -14.6)	<.001	-5.0 (-6.7, -3.3)	<.001
Work Ability Score baseline (NRS 0-10)	2.7 (2.3, 3.1)	<.001	1.2 (0.4, 2.0)	.004	-9.4 (-10.7, -8.0)	<.001	-2.9 (-5.4, -0.5)	.02	-14.3 (-15.6, -12.9)	<.001	-0.4, -2.3, 1.4)	.6
Widespread pain												
No	Ref.		Ref.		Ref.		Ref.		Ref.		Ref.	
Yes	1.09 (-2.0, 4.2)	.5	5.2 (1.7, 8.7)	.004	19.5 (8.9, 30.1)	<.001	10.9 (0.1, 21.7)	.05	22.2 (10.9, 33.4)	<.001	-8.9 (-16.9, -0.8)	.03
Anxiety/depression											–	–
No and slight	Ref.		Ref.		Ref.		Ref.		Ref.		–	–
Moderate to extreme	-4.8 (-7.5, -2.1)	.001	–		27.1 (17.9, 36.3)	<.001	11.8 (1.4, 22.2)	.03	10.0 (0.03, 20.0)	.05	–	–
Education			–									
≤12 y	Ref.		–		Ref.		Ref.		Ref.		–	–
≥13 y	1.9 (-0.4, 4.4)	.2	–		-18.2 (-26.5, -9.9)	<.001	-17.9 (-27.2, -8.6)	<.001	-19.7 (-28.6, -10.8)	<.001	–	–
Occupational rehabilitation											–	–
No	Ref.		Ref.		Ref.		Ref.		Ref.		–	–
Yes	1.9 (-0.8, 4.7)	.2	-4.1 (-8.0, -0.2)	.04	44.4 (35.1, 53.7)	<.001	13.7 (1.7, 25.7)	.03	-52.7 (-62.6, -42.7)	<.001	–	–
Sick leave in the year before rehabilitation	0.05 (0.04, 0.08)	<.001	-0.009 (-0.02, 0.04)	.6	0.4 (0.3, 0.4)	<.001	0.3 (0.2, 0.4)	<.001	-0.06 (-0.7, -0.5)	<.001	-0.04 (-0.1, 0.03)	.2
Work assessment allowance in the year before rehabilitation	-0.06 (-0.07, -0.05)	<.001	-0.05 (-0.07, -0.03)	<.001	0.3 (0.2, 0.3)	<.001	0.1 (0.034, 0.2)	.002	0.2 (0.3, 0.2)	<.001	0.3 (0.3, 0.4)	<.001
Disability benefits in the year before rehabilitation	-0.04 (-0.6, -0.04)	<.001	-0.04 (-0.06, -0.02)	<.001	-0.3 (-0.4, -0.3)	<.001	-0.4 (-0.4, -0.3)	<.001	0.9 (0.8, 0.9)	<.001	0.9 (0.8, 0.9)	<.001

NRS, numeric rating scale; Ref., reference category.

Only variables significant in both bivariate and final multivariate models are shown.

Variables with *P* < .05 are left in the models.

a Multiple linear regression with backward selection, adjusted for age, gender, geographic region, comorbidities, and diagnoses. The benefits are analysed in separate models.

Examining each health-related benefit separately, a 1-point improvement in work ability at 3 months after rehabilitation admission was associated with changes in benefit use during the following year. It corresponded to 1.5 additional sick leave days (95% CI: 0.8, 2.3), 9.5 fewer days on WAA (95% CI: −11.9, −7.2), and 5 fewer days on disability benefits (95% CI: −6.7, −3.3) in the year after rehabilitation (year 1) (Table 3).

The variance explained (R^2^) by the multivariate models varied across outcomes: 14% for sick leave, 33% for WAA, and 66% for disability benefits.

We examined associations with work ability at 12 months after rehabilitation admission and found results similar to those at 3 months for total days on benefits (−16.2 days [95% CI: -17.5, -14.9], *P* < .001) (Supplementary Table S1), WAA (−10.4 days [95% CI: −12.2, −8.7], *P* < .001) (Supplementary Table S2) and disability benefits (−6.3 days [95% CI: −7.9, −4.5], *P* = .006) (Supplementary Table S3). However, work ability at 12 months was not significantly associated with sick leave (0.04 days [95% CI: -0.5, 0.6], *P* = .9) (Supplementary Table S4).

## DISCUSSION

The present study indicates that higher levels of work ability at 3 and 12 months after rehabilitation admission were associated with fewer days on WAA and disability benefits in the following year (year 1). In contrast, a higher number of sick leave days was associated with higher work ability at 3, but not at 12 months.

Previous research in working-age populations supports our findings on long-term benefit use. Lower levels of work ability have been associated with prolonged use of health-related benefits such as disability pension and early retirement [[17], [18], [19],^37^]. This suggests that work ability is a useful indicator of future reliance on long-term benefits. Most evidence comes from general population cohorts, whereas studies in rehabilitation settings are fewer and often based on small samples. Our study adds to the literature by demonstrating similar associations in a large rehabilitation population, where patients commonly present with complex challenges related to work participation, comorbidities, and higher prior benefit use. In such settings, baseline work ability has previously been shown to predict applications for disability pension among people with back pain [38]. In addition, higher baseline work ability, as well as improvements following vocational rehabilitation, have been linked to successful return to work [39].

In contrast to some previous studies [16,17,19,37], we found that higher work ability at 3 months after rehabilitation admission (year 0) was associated with more days on sick leave, whereas no such association was observed at 12 months. This may reflect that we did not distinguish between short- and long-term sick leave. Previous research suggests that shorter absence from work prior to the rehabilitation intervention predicts return to work (RTW), and that long-term sick leave is associated with a reduced likelihood of RTW [40,41]. In this cohort of people with chronic or long-term conditions, some may anticipate a decline in health or transition to more permanent benefits as their sick leave period approaches its end after 52 weeks. Those on sick leave, particularly early in their leave, remain more closely connected to working life, as sick leave can function as a strategy to maintain employment while managing health issues [42], and may therefore report good work ability despite being absent. This is consistent with evidence linking stronger return-to-work beliefs to higher work ability [43], and may also explain the relatively low explained variance in the sick leave model, given the likely influence of unmeasured workplace-related factors [34].

We explored whether the association between work ability and benefit use differed at 3 and 12 months after rehabilitation admission (year 0). Changes in work ability in the rehabilitation year were examined in a previous study [20]. Returning to work or transitioning to long-term benefits is a complex process. It often involves multiple shifts between work and different benefits used over time [44], which may lead to changes in work ability. Work ability at 3 months may reflect that participants have not yet experienced the full effects of rehabilitation or implemented intended changes. On the other hand, by 12 months, in addition to rehabilitation, workplace and psychosocial factors such as supervisor support, job demands, and motivation may also play a role in shaping outcomes [45]. Exploring the association with work ability at both timepoints allows for capturing both immediate and long-term influences. Work ability at both 3 and 12 months was strongly associated with the use of long-term benefits in the following year (year 1). These findings suggest that monitoring work ability in the months after rehabilitation may help identify people more likely to rely on long-term benefits and guide targeted support.

Occupation did not independently contribute to the final multivariate models for any outcome. This contrasts with studies linking physically demanding work to disability in people with RMDs [46], although findings are inconsistent. Work participation is often associated with better health, wellbeing, and quality of life, highlighting the importance of timely, tailored support to help people with RMDs enter, remain in, or return to work [3,9,46].

Occupational rehabilitation was associated with more days on benefits in the year following rehabilitation (year 1) compared with the multidisciplinary rehabilitation programme. Participants in this group also had more days on WAA during the rehabilitation year (year 0), and more days of sick leave before rehabilitation. This suggests that referrals are relatively late in their trajectory, which may limit the potential for improvement [41,47]. These patterns contrast with studies reporting favourable return-to-work outcomes and cost-effectiveness of occupational rehabilitation [[48], [49], [50], [51]]. Notably, these earlier studies were conducted among people on long-term sick leave, whereas in our sample, participants in occupational rehabilitation had more days on WAA than on sick leave. People on WAA face more complex and prolonged work participation barriers, which may partly explain the lack of similar improvements. Taken together, the higher benefit use in our study likely reflects a more disadvantaged starting point rather than poorer rehabilitation outcomes. Nonetheless, since occupational rehabilitation is designed to aid return to work for people on both WAA and long-term sick leave, greater reductions in benefit use than what we found might have been expected. This finding warrants further exploration in future research.

Strengths of our study include the large, multicentre cohort, providing a representative sample of rehabilitation participants. Second, data were collected prospectively at multiple time points, supporting the interpretation of associations. Third, combining work ability with registry-based health-related benefit data allowed for a comprehensive dataset. This ensured a complete, valid and reliable measurement of outcomes while avoiding recall bias. Finally, the 3-year observation period, including the year before rehabilitation, the rehabilitation year, and the year after rehabilitation, allowed examination of patterns in benefit used over time.

Some limitations of our study must be considered. First, findings should be viewed in the context of Norway’s affluent welfare system, which provides benefits more easily than in other countries. Second, we used the WAS to assess work ability, which has not been formally validated in Norwegian but was adapted from the Swedish version. Although Norway and Sweden share closely related languages, comparable healthcare systems, and similar patient populations, subtle linguistic and cultural differences may not be fully captured without formal psychometric validation. We also did not include workplace factors, which are known to play an important role in the return-to-work process [45]. In addition, work participation was operationalised through the use of health-related benefits, and outcomes such as job loss were not included. Third, the patient group was heterogeneous with respect to diagnoses and rehabilitation programmes, which increases the generalisability but may mask diagnosis- or intervention-specific patterns. The referral diagnosis was coded according to the ICD-10, which reflects the primary medical condition but may not fully capture the severity or functional impact underlying the need for rehabilitation. We could not adjust for duration in the analyses because of extensive missing data for the date of discharge. Most of the participants were female, which may reflect that more women than men are attending rehabilitation, and women are more likely to participating in clinical studies than men [52,53]. Finally, fatigue, often associated with widespread pain and reduced work ability, was not included as a variable.

Future research could apply a longer follow-up period to capture the full trajectory of health-related benefit use with transitions between different benefits. Although WAA is typically granted for up to 3 years, our data suggest that its use begins to decline within the first year following rehabilitation. Thus, a longer observation period would be needed to determine how it evolves over the full entitlement period.

In summary, higher self-reported work ability 3 and 12 months after admission to rehabilitation was positively associated with subsequent work participation, measured by health-related benefit use in the following year, and adjusted for baseline health and demographic factors. These findings suggest that work ability may be a useful marker in heterogeneous rehabilitation cohorts, not only for identification of a need for WAA or disability benefits but also more broadly as an indicator for future work participation or labour market exclusion.

## CRediT authorship contribution statement

**Mari Nilsen Skinnes:** Writing – review & editing, Writing – original draft, Visualization, Validation, Supervision, Software, Resources, Project administration, Methodology, Investigation, Funding acquisition, Formal analysis, Data curation, Conceptualization. **Till Uhlig:** Writing – review & editing, Writing – original draft, Visualization, Validation, Supervision, Software, Resources, Project administration, Methodology, Investigation, Funding acquisition, Formal analysis, Data curation, Conceptualization. **Thomas Johansen:** Writing – review & editing, Writing – original draft, Visualization, Validation, Supervision, Software, Resources, Project administration, Methodology, Investigation, Funding acquisition, Formal analysis, Data curation, Conceptualization. **Idun Eid:** Writing – review & editing, Writing – original draft, Visualization, Validation, Supervision, Software, Resources, Project administration, Methodology, Investigation, Funding acquisition, Formal analysis, Data curation, Conceptualization. **Hanne Ludt Fossmo:** Writing – review & editing, Writing – original draft, Visualization, Validation, Supervision, Software, Resources, Project administration, Methodology, Investigation, Funding acquisition, Formal analysis, Data curation, Conceptualization. **Andreas Habberstad:** Writing – review & editing, Writing – original draft, Visualization, Validation, Supervision, Software, Resources, Project administration, Methodology, Investigation, Funding acquisition, Formal analysis, Data curation, Conceptualization. **Katerina Kandlova:** Writing – review & editing, Writing – original draft, Visualization, Validation, Supervision, Software, Resources, Project administration, Methodology, Investigation, Funding acquisition, Formal analysis, Data curation, Conceptualization. **Ingvild Kjeken:** Writing – review & editing, Writing – original draft, Visualization, Validation, Supervision, Software, Resources, Project administration, Methodology, Investigation, Funding acquisition, Formal analysis, Data curation, Conceptualization. **Tarja Rajalahti Kvalheim:** Writing – review & editing, Writing – original draft, Visualization, Validation, Supervision, Software, Resources, Project administration, Methodology, Investigation, Funding acquisition, Formal analysis, Data curation, Conceptualization. **Peter Solvoll Lyby:** Writing – review & editing, Writing – original draft, Visualization, Validation, Supervision, Software, Resources, Project administration, Methodology, Investigation, Funding acquisition, Formal analysis, Data curation, Conceptualization. **Ross Wilkie:** Writing – review & editing, Writing – original draft, Visualization, Validation, Supervision, Software, Resources, Project administration, Methodology, Investigation, Funding acquisition, Formal analysis, Data curation, Conceptualization. **Nina Østerås:** Writing – review & editing, Writing – original draft, Visualization, Validation, Supervision, Software, Resources, Project administration, Methodology, Investigation, Funding acquisition, Formal analysis, Data curation, Conceptualization. **Rikke Helene Moe:** Writing – review & editing, Writing – original draft, Visualization, Validation, Supervision, Software, Resources, Project administration, Methodology, Investigation, Funding acquisition, Formal analysis, Data curation, Conceptualization.

## Competing interests

All authors declare they have no competing interests.

## Appendix Supplementary materials

Image, application 1

Image, application 2

## Footnotes

Supplementary material associated with this article can be found in the online version at doi:10.1016/j.ero.2026.03.011.
