# Supplementary material for: Associations between work ability and work participation after rehabilitation: a longitudinal multicentre cohort study
Source: EULAR Rheumatol Open. 2026 Apr 17;2(2):100163. doi: 10.1016/j.ero.2026.03.011 (PMC13425225; doi:10.1016/j.ero.2026.03.011)
Supplement: Supplementary file 1 [file mmc1.docx]

Supplementary materials for: Associations between work ability and work participation after rehabilitation – a longitudinal multicentre cohort study

Textbox: Brief description of the rehabilitation programmes

*Multidisciplinary rehabilitation:*

- Targets people with complex or long-term health conditions
- Offered across specialized institutions, each serving specific patient populations
- Based on goal-oriented communication and functional assessments
- Combines individual- and group sessions
- Delivered by multidisciplinary teams: physiotherapists, occupational therapists, nurses, physicians, psychologists, social workers, exercise specialists and vocational counsellors
  - Aims to promote functioning, coping and participation; return to work (RTW) can be a key goal for working-age people

*In addition to above, occupational rehabilitation:*

- Targets people who are sick-listed or have reduced work ability
- Primary goal: RTW
- Key components: Work ability assessments, work-focused cognitive strategies, development of RTW-planning, self-management education, workplace communication, involvement of an RTW coordinator

Days on benefits for the occupational and the multidisciplinary rehabilitation groups.


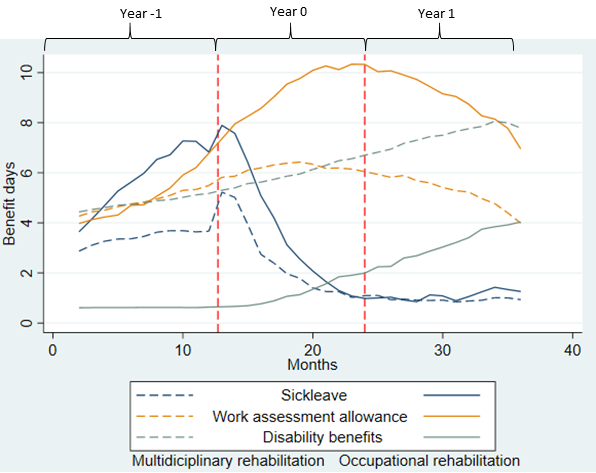

Supplementary Figure S1. Days with benefits per month during the three-year period, for the multidisciplinary and the occupational rehabilitation groups.

Sick leave increased more for the occupational rehabilitation group than for the multidisciplinary rehabilitation group at the onset of rehabilitation (year 0), and declined to the same level for both groups at month 22 (year 0). WAA reached its peak for the occupational rehabilitation group at approximately the same time as the sick leave reached bottom at month 22, while the multidisciplinary rehabilitation group utilized less days of WAA. Disability benefits increased gradually for both groups during the three-year period, but the multidisciplinary rehabilitation group had more days of disability benefits than the occupational rehabilitation group (Figure S1, Figure S2).


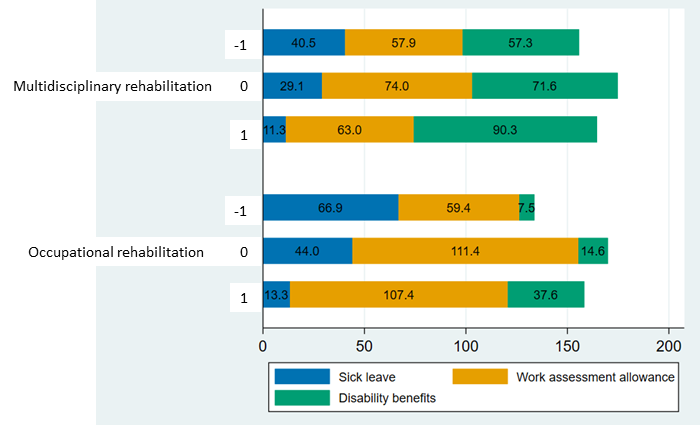
 Supplementary Figure S2. Average days on benefits per year during the three-year period, for the multidisciplinary and the occupational rehabilitation groups.


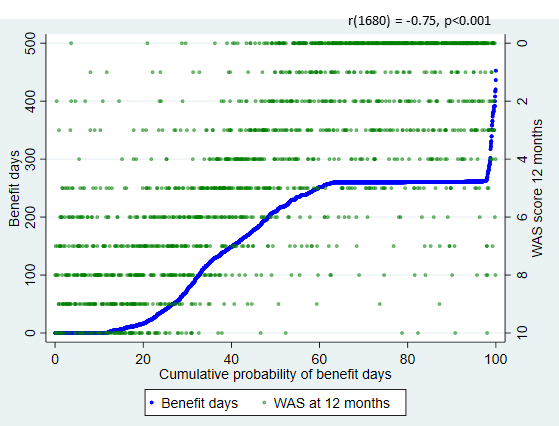


Supplementary Figure S3. Relationship between work ability score at 12 months after rehabilitation admission (year 0) and number of benefit days in the year after rehabilitation (year 1) in a cumulative probability plot.

Supplementary Table S1. Associations between benefit days in the year after rehabilitation (year 1) and work ability at 12 months after the start of rehabilitation (year 0), multiple linear regression. Adjusted for age, gender, geographic region comorbidities and diagnosis.

| Variable | Univariate model  β, (95%CI) | p-value | Multivariate model β, (95 % CI) R^2^= 0.68 | p-value |
| --- | --- | --- | --- | --- |
| Work ability score 12 months (0-10) | -24.6 (-25.6, -23.5) | <0.001 | -16.2 (-17.5, -14.9) | <0.001 |
| Work ability score baseline (0-10) | -21.0 (-22.2, -19.7) | <0.001 | -3.9 (-5.4, -2.4) | <0.001 |
| Smoking |  |  | - |  |
| Never | Ref. |  | - |  |
| Sometimes | 15.3 (6.1, 24.6) | 0.001 | - |  |
| Daily | 32.5 (17.0, 47.9) | <0.001 | - |  |
| BMI | -1.6 (-2.3, -1.0) | <0.001 | - |  |
| Education |  |  |  |  |
| ≤ 12 years | Ref. |  | Ref. |  |
| ≥ 13 years | -35.8 (-44.5, -27.1) | <0.001 | -12.6 (-20.7, -4.6) | 0.002 |
| Occupation |  |  |  |  |
| Manual occupations | Ref. |  | Ref. |  |
| Professional/manager | -33.1 (-42.1, -24.1) | <0.001 | -8.3 (-16.3, -0.2) | 0.04 |
| Widespread pain |  |  | - |  |
| No | Ref. |  | - |  |
| Yes | 42.8 (32.1, 53.5) | <0.001 | - |  |
| Pain intensity (0-10) | 10.4 (7.7, 13.1) | <0.001 | - |  |
| Anxiety/depression |  |  | - |  |
| No and Slightly | Ref. |  | - |  |
| Moderate to Extreme | 32.3 (22.5, 42.1) | <0.001 | - |  |
| Occupational rehabilitation |  |  |  |  |
| No | Ref. |  | Ref. |  |
| Yes | -6.4 (-16.3, 3.6) | 0.2 | - |  |
| Data collection during covid-19 |  |  | - |  |
| No | Ref. |  | - |  |
| Yes | -2.4 (-11.8, 7.1) | 0.6 | - |  |
| Sick-leave in the year prior to rehab | -0.2 (-0.3, -0.1) | <0.001 | 0.2 (0.2, 0.3) | <0.001 |
| Work assessment allowance | 0.4 (0.4, 0.4) | <0.001 | 0.3 (0.3, 0.4) | <0.001 |
| Disability benefits | 0.5 (0.4, 0.5) | <0.001 | 0.4 (0.3, 0.4) | <0.001 |

CI: Confidence Interval. BMI: Body Mass Index.

Supplementary Table S2. Associations between days on work assessment allowance in the year after rehabilitation (year 1) and work ability at 12 months after the start of rehabilitation, multiple linear regression. Adjusted for age, gender, geographic region comorbidities and diagnosis.

| Variable | Univariate model  β, (95%CI) | p-value | Multivariate model β (95 % CI) R^2^= 0.37 | p-value |
| --- | --- | --- | --- | --- |
| Work ability score 12 months (0-10) | -10.8 8-12.2, -9.4) | <0.001 | -10.4 (-12.2, -8.7) | <0.001 |
| Work ability score baseline (0-10) | -9.4 (-10.7, -8.0) | <0.001 | -5.5 (-7.5, -3.5) | <0.001 |
| Smoking |  |  |  |  |
| Never | Ref. |  | - |  |
| Sometimes | -3.5 (-12.3, 5.3) | 0.4 | - |  |
| Daily | 3.8 (-10.8, 18.5) | 0.6 | - |  |
| BMI | -0.9 (-1.5, -0.3) | 0.003 | - |  |
| Education |  |  | - |  |
| ≤ 12 years | Ref. |  | Ref. |  |
| ≥ 13 years | -18.2 (-26.5, -9.9) | <0.001 | -15.5 (-24.1, -6.9) | <0.001 |
| Occupation |  |  | - |  |
| Manual occupations | Ref. |  | - |  |
| Professional/manager | -14.4 (-22.9, -5.9) | 0.001 | - |  |
| Widespread pain |  |  | - |  |
| No | Ref. |  | - |  |
| Yes | 19.5 (8.9, 30.1) | <0.001 | - |  |
| Pain intensity (0-10) | 6.5 (3.9, 9.0) | <0.001 | - |  |
| Anxiety/depression |  |  | - |  |
| No and Slightly | Ref. |  | - |  |
| Moderate to Extreme | 27.1 (17.9, 36.3) | <0.001 | - |  |
| Occupational rehabilitation |  |  |  |  |
| No | Ref. |  | Ref. |  |
| Yes | 44.4 (35.1, 53.7) | <0.001 | 15.5 (3.5, 27.4) | 0.01 |
| Data collection during covid-19 |  |  |  |  |
| No | Ref. |  | - |  |
| Yes | 12.6 (3.6, 21.5) | 0.006 | - |  |
| Sick-leave in the year prior to rehab | 0.4 (0.3, 0.4) | <0.001 | 0.3 (0.2, 0.3) | <0.001 |
| Work assessment allowance | 0.3 (0.2, 0.3) | <0.001 | 0.04 (-0.2, 0.1) | 0.2 |
| Disability benefits | -0.3 (-0.4, -0.3) | <0.001 | -0.4 (-0.5, -0.4) | <0.001 |

CI: Confidence Interval. BMI: Body Mass Index.

Supplementary Table S3. Associations between days on disability benefits in the year after rehabilitation (year 1) and work ability at 12 months after rehabilitation start, multiple linear regression. Adjusted for age, gender, geographic region comorbidities and diagnosis.

| Variable | Univariate model  β, (95%CI) | p-value | Multivariate model β (95 % CI) R^2^= 0.68 | p-value |
| --- | --- | --- | --- | --- |
| Work ability score 12 months (0-10) | -15.6 (-17.0, -14.2) | <0.001 | -6.3 (-7.9, -4.5) | 0.006 |
| Work ability score baseline (0-10) | -14.3 (-15.6, -12.9) | <0.001 | -0.6 (-2.4, 1.1) | 0.5 |
| Smoking |  |  |  |  |
| Never | Ref. |  | - |  |
| Sometimes | 18.5 (9.2, 27.9) | <0.001 | - |  |
| Daily | 31.4 (15.9, 47.1) | <0.001 | - |  |
| BMI | -0.9 (-1.5, -0.3) | 0.005 | - |  |
| Education |  |  | - |  |
| ≤ 12 years | Ref. |  | - |  |
| ≥ 13 years | -19.7 (-28.6, -10.8) | <0.001 | - |  |
| Occupation |  |  |  |  |
| Manual occupations | Ref. |  | Ref. |  |
| Professional/manager | -21.8 (-30.9, -12.7) | <0.001 | - |  |
| Widespread pain |  |  |  |  |
| No | Ref. |  | Ref. |  |
| Yes | 22.2 (10.9, 33.4) | <0.001 | -12.0 (-20.6, -3.5) | 0.006 |
| Pain intensity (0-10) | 4.1 (1.4, 6.9) | 0.004 | - |  |
| Anxiety/depression |  |  |  |  |
| No and Slightly | Ref. |  | - |  |
| Moderate to Extreme | 10.0 (0.03, 20.0) | 0.05 | - |  |
| Occupational rehabilitation |  |  | - |  |
| No | Ref. |  | - |  |
| Yes | -52.7 (-62.6, -42.7) | <0.001 | - |  |
| Data collection during covid-19 |  |  | - |  |
| No | Ref. |  | - |  |
| Yes | -15.0 (-24.6, -5.4) | 0.002 | - |  |
| Sick-leave in the year prior to rehab | -0.06 (-0.7, -0.5) | <0.001 | -0.04 (-0.1, 0.02) | 0.1 |
| Work assessment allowance | 0.2 (0.3, 0.2) | <0.001 | 0.3 (0.3, 0.4) | <0.001 |
| Disability benefits | 0.9 (0.8, 0.9) | <0.001 | 0.8 (0.8, 0.9) | <0.001 |

CI: Confidence Interval. BMI: Body Mass Index.

Supplementary Table S4. Associations between sick leave days in the year after rehabilitation (year 1) and work ability at 12 months after the start of rehabilitation, multiple linear regression. Adjusted for age, gender, geographic region comorbidities and diagnosis.

| Variable | Univariate model  β, (95%CI) | p-value | Multivariate model β, (95 % CI) R^2^= 0.11 | p-value |
| --- | --- | --- | --- | --- |
| Work ability score 12 months (0-10) | 1.9 (1.4, 2.3) | <0.001 | 0.04 (-0.5, 0.6) | 0.9 |
| Work ability score baseline (0-10) | 2.66 (2.3, 3.1) | <0.001 | 1.9 (1.2, 2.6) | <0.001 |
| Smoking |  |  |  |  |
| Never | Ref. |  | Ref. |  |
| Sometimes | 0.2 (-2.3, 2.8) | 0.9 | - |  |
| Daily | -2.8 (-7.1, 1.5) | 0.2 | - |  |
| BMI | 0.1 (-0.5, 0.3) |  | - |  |
| Education |  |  | - |  |
| ≤ 12 years | Ref. |  | - |  |
| ≥ 13 years | 1.9 (-0.4, 4.4) | 0.2 | - |  |
| Occupation |  |  |  |  |
| Manual occupations | Ref. |  | Ref. |  |
| Professional/manager | 3.1 (0.6, 5.6) | 0.02 | - |  |
| Widespread pain |  |  |  |  |
| No | Ref. |  | Ref. |  |
| Yes | 1.09 (-2.0, 4.2) | 0.5 | - |  |
| Pain intensity (0-10) | -0.2 (-0.9, 0.5) | 0.6 | - |  |
| Anxiety/depression |  |  |  |  |
| No and Slightly | Ref. |  | - |  |
| Moderate to Extreme | -4.8 (-7.5, -2.1) | 0.001 | - |  |
| Occupational rehabilitation |  |  |  |  |
| No | Ref. |  | Ref. |  |
| Yes | 1.9 (-0.8, 4.7) | 0.2 | - |  |
| Data collection during covid-19 |  |  |  |  |
| No | Ref. |  | Ref. |  |
| Yes | 0.1 (-2.5, 2.7) | 0.9 | - |  |
| Sick-leave in the year prior to rehab | 0.05 (0.04, 0.08) | <0.001 | 0.004 (-0.02, 0.03) | 0.8 |
| Work assessment allowance | -0.06 (-0.07, -0.05) | <0.001 | -0.05 (-0.07, -0.02) | <0.001 |
| Disability benefits | -0.04 (-0.6, -0.04) | <0.001 | -0.04 (-0.07, -0.02) | <0.001 |

CI: Confidence Interval. BMI: Body Mass Index.
