# Supplementary material for: Associations between work ability and work participation after rehabilitation: a longitudinal multicentre cohort study
Source: EULAR Rheumatol Open. 2026 Apr 17;2(2):100163. doi: 10.1016/j.ero.2026.03.011 (PMC13425225; doi:10.1016/j.ero.2026.03.011)
Supplement: Supplementary file 2 [file mmc2.docx]

**PPI in the RehabNytte Study, GRIPP 2-SFa.**

| Section and topic | Item |
| --- | --- |
| 1: Aim  Report the aim of PPI in the study | This research is conducted in close collaboration with “The Norwegian Federation of Organisations of Disabled People (FFO), a national patient umbrella organization representing people with disabilities and chronic diseases.  The aim of involving patient research partners was to ensure that the research questions, outcome measures, and interpretation of results were relevant, meaningful, and grounded in users’ experiences. Patient involvement was integrated throughout the research process to strengthen the practical and societal relevance of the study. |
|  |  |
| 2: Methods  Provide a clear description of the  methods used for PPI in the study | Patient and public involvement was informed by established principles for involving patient research representatives in research, including the European League Against Rheumatism (EULAR) (1).  Patient research partners were involved at multiple stages of the project and had roles as advisors and co-researchers. They were recruited through FFO and from participating rehabilitation institutions to ensure diversity of perspectives and relevance to rehabilitation services.  Two actively engaged patient representatives were members of the project group from the planning phase onward. Their contributions included:   - Discussion of research aims and priorities - Feedback on selection of outcome measures and questionnaires - Reflection on analytical approaches and interpretation of findings - Wording and contextualisation of the results - Contribution to dissemination of results   Roles and expectations were clarified early in the collaboration process, with patient partners recognised as co-researchers, co-authors and collaborators contributing equally alongside academic expertise. Communication took place through meetings, e-mails, and telephone calls as appropriate.  The project was funded through the DAM Foundation, which supports research in collaboration with patient organisations. |
|  |  |
| 3: Results  Outcomes—Report the results of  PPI in the study, including both  positive and negative outcomes | PPI contributed to the study in several ways, including:   - Interpretation of results - Added important viewpoints to discussions of the results - Writing of the manuscripts |
|  |  |
| 4: Discussion  Outcomes—Comment on the extent to  which PPI influenced the study overall.  Describe positive and negative effects | Patient and public involvement had a meaningful influence on the design and interpretation of the study. The collaboration strengthened the relevance of the results, the direction of the discussion, and supported a more nuanced understanding of rehabilitation from a service user perspective.  Not all suggestions from the patient research partners could be fully implemented, due to predefined analytical frameworks. |

**References:**

1. de Wit, M.P., et al., European League Against Rheumatism recommendations for the inclusion of patient representatives in scientific projects. Ann Rheum Dis, 2011. 70(5): p. 722-
